# Cognitive Sequelae of Central-Variant Posterior Reversible Encephalopathy Syndrome (PRES)

**DOI:** 10.1155/2021/8850316

**Published:** 2021-02-05

**Authors:** Joseph Seemiller, Muhammad Taimur Malik

**Affiliations:** ^1^Geisinger Neuroscience Institute, Danville, PA, USA; ^2^Geisinger Commonwealth School of Medicine, Scranton, PA, USA

## Abstract

**Introduction:**

Although the posterior reversible encephalopathy syndrome (PRES) is often associated with headache and visual changes, central-variant PRES can be difficult to clinically diagnose in a patient with alteration of consciousness. Central-variant PRES has been previously described in the literature affecting subcortical white matter and the brainstem. *Case Presentation*. We describe a case presenting with hypertension (192/98) and altered level of consciousness requiring intubation. She was ultimately found to have extensive symmetric cortical and subcortical edema, with extensive involvement of bilateral thalami, consistent with central-variant PRES. Her mentation rapidly improved with blood pressure management. Confirmation of the diagnosis of central-variant PRES was made on repeat brain imaging. Our case is unique in demonstrating dramatic central white matter changes and their reversibility on repeat imaging six days later. Finally, persistent cognitive deficits at follow-up four months later are described.

**Conclusion:**

Atypical presentations of PRES, involving alterations in levels of consciousness, can be difficult to clinically diagnose. A thorough differential diagnosis is even more important in cases of PRES with atypical imaging. Recognition of the diagnostic patterns of PRES on brain imaging, with prompt reversal of the causative factors, is crucial for the appropriate care of these patients. The long-term sequelae, which could include cognitive deficits, are poorly studied and understood.

## 1. Introduction

Posterior reversible encephalopathy syndrome (PRES) refers to a reversible neurologic syndrome with symptoms including headache, changes in mental status, visual changes, and seizures. There are numerous causes of PRES including hypertension, immunosuppressive and antineoplastic medications, renal failure, autoimmune disorders, and many other causes [1–3]. Imaging shows subcortical and cortical white matter edema which is classically parieto‐occipital predominant [4], however, less commonly can involve the brainstem, basal ganglia, and cerebellum [5].

We describe a case of central-variant PRES who presented atypically with alteration in level of consciousness. She had changes on magnetic resonance imaging (MRI) brain involving regions associated with atypical PRES, including changes in the subcortical white matter in the cerebellum and cerebrum, basal ganglia, and extensive involvement of bilateral thalami. The diagnosis was confirmed with an extensive workup including lumbar puncture, ultimately with confirmation of central-variant PRES diagnosis through repeat MRI brain. This case demonstrates the importance of prompt recognition of atypical or central-variant PRES and the differential diagnosis of atypical PRES, and further, provides impressive imaging findings consistent with central-variant PRES. Finally, the long-term cognitive deficits experienced by this patient demonstrate, with a discussion of literature, that the chronic sequelae of PRES are poorly understood and can include cognitive changes.

## 2. Case Report

A 59-year-old woman presented to a community hospital with two days of headache and vomiting followed by acute development of somnolence. On hospital presentation, late on day two after symptom onset, her initial blood pressure was 192/98 and temperature 38.0°C. Levetiracetam 500 mg twice daily was started despite no reported history of convulsions. She was then transferred to a tertiary care hospital in central Pennsylvania. Physical exam was remarkable for lower extremity hyper-reflexia and Babinski sign bilaterally and was otherwise nonfocal.

Empiric treatment for meningitis was initiated with ceftriaxone, vancomycin, acyclovir, and ampicillin. Antibiotics were stopped after lumbar puncture revealed 3 WBC/mm^3^, 69 mg/dL protein, and 83 mg/dL glucose on day three after symptom onset.

**Figure 1:**
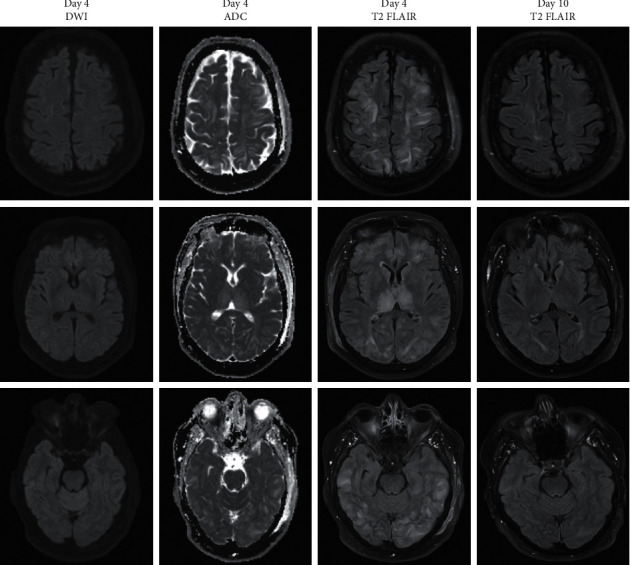
Sequences of MRI brain including DWI, ADC, initial FLAIR, and FLAIR on follow-up imaging, from left to right. Initial MRI (first three columns on the left) noted symmetric subcortical and cortical FLAIR hyperintensity involving the bilateral cerebral hemispheres including extensive involvement of the thalami and basal ganglia, without contrast enhancement. DWI was isointense, lacking diffusion restriction, and ADC was hyperintense, consistent with vasogenic edema. FLAIR sequence on repeat MRI (fourth column from the left) noted significant interval improvement, with mild residual hyperintensity in the parieto-occipital white matter.

On day four after symptom onset, MRI brain was obtained, showing bilateral subcortical FLAIR hyperintensities without diffusion restriction, consistent with vasogenic edema (Figure 1). Aggressive blood pressure control was initiated with a goal <140/90.

Low sensitivity C-reactive protein (CRP) was elevated at 22 mg/L and albumin was normal at 4.8 g/dL. Vitamin B12 was <150pg/mL, methylmalonic acid was elevated at 323 nmol/L, and homocysteine was elevated at 17.7 umol/L. Vitamin B12 supplementation was started with 1 mg intramuscular daily for seven days, followed by oral daily dosing of 1 mg on an ongoing basis. Thiamine was empirically supplemented with 500 mg intravenous daily for three days, after which 100 mg oral daily dosing was continued. However, whole-blood thiamine taken on admission was ultimately found to be normal at 105 nmol/L. Urine toxicology testing was significant for marijuana. Infectious studies including HIV antibody and antigen, lyme antibody, West Nile panel, arbovirus panel, anaplasma polymerase chain reaction (PCR), Babesia PCR and enterovirus PCR, and peripheral blood smear were negative. Additional studies including antinuclear antibody, antineutrophil cytoplasmic antibody, folate, copper, and peripheral blood smear were negative.

Ten days after symptom onset, repeat MRI brain showed significant improvement of white matter hyperintensities, and MRI cervical spine noted no myelopathy. Central-variant PRES was diagnosed based on rapid reversibility of white matter lesions with blood pressure control and lack of myelopathy to suggest symptomatic depletion of vitamin B12.

At follow-up four months after the initial hospitalization, the patient reported persistent cognitive impairments since her hospitalization, involving short-term memory and attention deficits. A Montreal Cognitive Assessment (MoCA) was 21/30 with errors including 1/30 for cube drawing (visuospatial), 1/30 for reading digits and 1/30 for tapping on a specified letter (attention), 2/30 for repetition and 1/30 for fluency (language), 2/30 for delayed recall, and 1/30 for orientation. She declined formal neuropsychological assessment.

## 3. Discussion

This patient's presentation, with an acute to subacute onset of headache, fever, and vomiting, with progression to somnolence, prompted an initially broad differential diagnosis. MRI brain was instrumental for the identification of central-variant PRES. The imaging features leading to this diagnosis included symmetric subcortical hyperintensity on fluid attenuated inversion recovery (FLAIR) sequences, without diffusion restriction or contrast enhancement.

### 3.1. Differential Diagnosis

A thorough differential diagnosis is important in evaluation for PRES, especially for PRES with atypical imaging characteristics, as the reversibility of the clinical and radiographic features of PRES requires time to confirm and might not even be entirely reversible. MRI brain imaging is vital for early workup of suspected PRES.

Vascular diagnostic considerations could include reversible cerebral vasoconstriction syndrome, cerebral venous sinus thrombosis, and central nervous system vasculitis, for which additional vascular imaging could be obtained if suspected. Infectious considerations should include encephalitis and meningitis. Autoimmune mimics could include ADEM, lupus cerebritis, or autoimmune encephalitis. Suspected infectious or autoimmune etiology should prompt CSF workup, and pleocytosis would support an underlying infection or inflammatory etiology. Toxic leukoencephalopathy, such as that caused by inhaled heroin, should be considered through correlate social history and urine drug toxicology testing. Acute hepatic encephalopathy could be considered with hepatic function serology. Neoplastic causes such as lymphoma and gliomatosis cerebri could be suggested with reported B-symptoms, weight loss, and presence of enhancement on MRI. Specific considerations for central PRES include osmotic demyelination syndrome, acute hepatic encephalopathy, and hypoxic ischemic injury.

Diagnosis of PRES is limited by the lack of specific diagnostic criteria, even though several criteria have been proposed based on retrospective cohort studies, and approaches have been proposed for evaluation of suspected PRES. Typical PRES can be evaluated through an algorithm in >90% of patients presenting with parieto-occipital or a posterior frontal cortical-subcortical pattern of vasogenic edema on FLAIR MRI sequences [6]. However, atypical PRES is not accounted for in this algorithm. A more recently proposed algorithm lists several differential diagnostic considerations for PRES, which should be carefully reviewed for all cases of atypical PRES imaging, or typical PRES imaging with poor reversibility of clinical or radiographic abnormalities [7].

In this patient, the possibility of myelopathy was considered given the patient's hyper-reflexia and vitamin B12 deficiency. However, cervical spine MRI was unremarkable, and ultimately, the patient's vitamin B12 deficiency was thought noncontributory to her presentation. Viral meningitis and tic-borne illness were also considered; however, lumbar puncture without pleocytosis made these possibilities unlikely. Ultimately, the patient rapidly improved clinically through her hospital course which, in conjunction with her repeat MRI showing resolved FLAIR hyperintensity, confirmed the diagnosis of central-variant PRES.

### 3.2. Etiology of PRES

Originally, PRES was described by Hinchey in 1996 [3]. Typical imaging findings have been identified as symmetric hemispheric vasogenic edema in the white matter extending to the cortex, best seen on FLAIR with the absence of diffusion restriction [4].

Hypertension is one of the most common identified causes of PRES. There are multiple proposed theories explaining how hypertension enacts these effects. Traditionally, the vasogenic theory has maintained that severe hypertension exceeds the autoregulatory limits of the brain, resulting in extravasation of the fluid and edema [8–10]. However, this does not account for the findings of PRES in patients with normotension.

An alternative explanation for PRES pathogenesis is that cytotoxic insults from chemokines or exogenous toxins, or inflammatory insults from t-cell activation and cytokine release, can cause vasoconstriction. Vasoconstriction could also reduce perfusion, leading to ischemia and vasogenic edema. Supporting this theory are the findings of vasoconstriction and vasculopathy on angiography studies [11] as well as hypoperfusion [12] seen in PRES patients.

### 3.3. Imaging

The pattern of edema in PRES is known to occur in a posterior-to-anterior fashion, with involvement of the parieto-occipital region most commonly. However, central-variant PRES can involve the basal ganglia or brainstem and even spare the parieto-occipital region [5]. Our report demonstrates remarkable symmetric FLAIR hyperintensity involving cortical and subcortical regions, including the cerebellum and basal ganglia, notably with extensive involvement of bilateral thalami.

### 3.4. Serology

Serological markers have been associated with outcomes in patients with PRES. Elevated CRP levels have been associated with increased in-hospital mortality [13] The CRP level in this patient was elevated at 22, and although she survived the hospitalization, her morbidity could be reflected in her elevated CRP level; elevated CRP could reflect inflammation-related instability of the blood-brain barrier and associated endothelial damage [14]. Low serum albumin levels have been observed in 70% of patients with PRES [15]; however, albumin was normal in this patient. CSF albuminocytologic dissociation, without pleocytosis, was seen in this patient, which is a finding seen in 70% of patients with PRES. CSF protein is directly associated with the extent and topographical distribution of cerebral edema [16, 17].

### 3.5. Prognosis

Neurologic sequelae can persist in patients with PRES. In a study of 31 patients with PRES receiving chemotherapy, 84% of subjects returned to baseline, requiring a range of one to 167 days after symptom onset [18]. Another study with 111 cases reported 17 cases with ongoing neurologic sequelae [19]. There have also been suggestions that MRI severity could correlate with clinical outcome, but this relationship requires further study [6, 15, 20].

Long-term cognitive outcomes after PRES have not been well characterized in literature. One case series documented the long-term cognitive outcomes in two patients with typical PRES. Neuropsychological testing revealed predominantly spatial perception tasks, as well as milder impairments in attention and memory [21]. The deficits, primarily visuospatial, were thought consistent with posterior cortical involvement, seen in typical patterns of PRES. Similarly, in follow-up interviews of 210 women with a history of eclampsia, 22% reported decreased concentration or difficulty with recall [22]. However, neuropsychological assessment was unable to discern an attention or executive function impairment in patients with a history of eclampsia [23].

The cognitive deficits in this patient were limited by incomplete neuropsychological assessment; however, deficits were broad across domains tested in the MoCA. In the cases presented by [21], the visuospatial predominant deficits were thought related to the specific posterior cortical areas affected by PRES. In contrast, the extensive cortical involvement in our patient does not clearly localize. However, this case further supports existing evidence that the long-term sequelae of PRES are poorly understood. Long-term follow-up screening and neuropsychological testing in more patients with both typical and atypical PRES syndromes are needed to understand if its cognitive sequelae are less “reversible” than its imaging findings.

## Data Availability

No datasets were generated or analyzed during this study.
